# The Role of Hydroxycinnamic Acid Amide Pathway in Plant Immunity

**DOI:** 10.3389/fpls.2022.922119

**Published:** 2022-06-22

**Authors:** Saifei Liu, Jincheng Jiang, Zihui Ma, Muye Xiao, Lan Yang, Binnian Tian, Yang Yu, Chaowei Bi, Anfei Fang, Yuheng Yang

**Affiliations:** ^1^College of Plant Protection, Southwest University, Chongqing, China; ^2^Committee on Agriculture and Rural Affairs of Yongchuan District, Chongqing, China; ^3^Analytical and Testing Center, Southwest University, Chongqing, China

**Keywords:** hydroxycinnamic acid amides, plant immunity, antimicrobial activities, cell wall, *p*-coumaric acid, ferulic acid

## Abstract

The compounds involved in the hydroxycinnamic acid amide (HCAA) pathway are an important class of metabolites in plants. Extensive studies have reported that a variety of plant hydroxycinnamamides exhibit pivotal roles in plant–pathogen interactions, such as *p*-coumaroylagmatine and ferulic acid. The aim of this review is to discuss the emerging findings on the functions of hydroxycinnamic acid amides (HCAAs) accumulation associated with plant defenses against plant pathologies, antimicrobial activity of HCAAs, and the mechanism of HCAAs involved in plant immune responses (such as reactive oxygen species (ROS), cell wall response, plant defense hormones, and stomatal immunity). However, these advances have also revealed the complexity of HCAAs participation in plant defense reactions, and many mysteries remain to be revealed. This review provides an overview of the mechanistic and conceptual insights obtained so far and highlights areas for future exploration of phytochemical defense metabolites.

## Introduction

Plants are well stocked with chemical defense compounds that function in protection against herbivores and pathogens (Gershenzon and Ullah, 2022). The global metabolic reprogramming is a common event in plant innate immunity. A large number of compounds are involved in the process of plant disease resistance. For example, pathogens stimulated the phenylpropanoid pathway (PPP) and lead to the synthesis of secondary metabolites. These compounds can provide protection against biotic and abiotic stresses in plants. Hydroxycinnamic acid amides (HCAAs) are widely distributed in plant secondary metabolites and are often referred to as one of the major phenylpropanoid metabolites (Herrmann, 1989).

Hydroxycinnamic acid amides have been described throughout the plant kingdom and accumulated in various organs, sometimes at high concentrations, especially in injured tissues (Bassard et al., 2010). HCAAs are purported to function in several growth and developmental processes, including tuberization, flower development, pollen wall formation, pollen health effects sexual differentiation, senescence, cell division, and stress responses (Facchini et al., 2002; Luo et al., 2009). HCAAs pathway is an important offshoot pathway of the PPP (Kim et al., 2021). The PPP accessory pathway involves the biosynthesis of HCAAs, which are polymers made from hydroxycinnamic acids (HCAs) and polyamines (PA; Facchini et al., 2002). HCAAs are synthesized through the condensation of various biogenic amines with HCAs *via* BAHD acyltransferase (Li et al., 2018). HCAAs are thought to be the final accumulation product of PA and aromatic amine metabolism, or as a form of cellular storage to regulate the metabolic pool of the two parent components (Bassard et al., 2010). HCAAs may be secreted into the apoplast space under the action of multidrug and toxin extrusion (MATE) transporters (Zeiss et al., 2021). The HCAAs tend to be present in the insoluble-bound form, as they were covalently bound to the arabinoxylans of the cell wall hemicellulose. Through dimerization and trimerization, the insoluble-bound HCAAs form an extensive network of cross-linkages that deter insect herbivory in stored grain, prevent pathogen penetration during the growing season, and impart plant tolerance to drought (Butts-Wilmsmeyer et al., 2020). Moreover, many studies have demonstrated that the biosynthesis of HCAAs and their subsequent polymerization in the plant cell play a vital role in response to pathogenic infections (Muroi et al., 2009). For instance, 24 h post *Pseudomonas syringae* pv. *tomato* (*Pst*) DC3000 and *Erwinia carotovora carotovora* infections, the content of HCAAs in *Arabidopsis thaliana* leaves was significantly increased (Macoy et al., 2022). Tyramine-derived HCAAs overproduced in plants may interfere with colonization of *Ralstonia solanacearum* by becoming incorporated into the blood vascular and perivascular cell walls, thereby restricting the movement of pathogens within the plant (Kashyap et al., 2022). Pretreatment with *p*-Coumaric acid results in an accumulation of hydroxycinnamic acid in soluble and cell wall-bound form, which protects against infection by *Xanthomonas campestris* pv. *Campestris* (Islam et al., 2018). With the development of high-throughput metabolomics technology, numerous studies have demonstrated that HCAAs are involved in plant responses to biotic stress. By using a comparative metabolomic approach, researchers identified defense-related biosynthetic pathways as affected in susceptible and resistant wheat cultivars, and HCAAs were found accumulated within 4–8 days of fungal infection in wheat (Seybold et al., 2020). In addition, experiments have demonstrated that HCAAs have antimicrobial activity (Kyselka et al., 2018).

In current knowledge, HCAAs are still a key class of secondary metabolites that were defined as biomarkers to measure plant resistance. In a previous review, the important role of HCAAs in plant defense against pathogens was also described in detail (Macoy et al., 2015). On this basis, we summarized the latest research on HCAs/HCAAs in recent years. This review paper aims to give a thorough understanding on the functions of HCAs/HCAAs accumulation during defense responses, antimicrobial activity of HCAs/HCAAs, mechanism of HCAs/HCAAs involved in plant immune responses, and the regulation of HCAAs biosynthesis in plants.

## The Biosynthesis of HCAAs From the Phenylpropanoid Pathway

**Figure 1:**
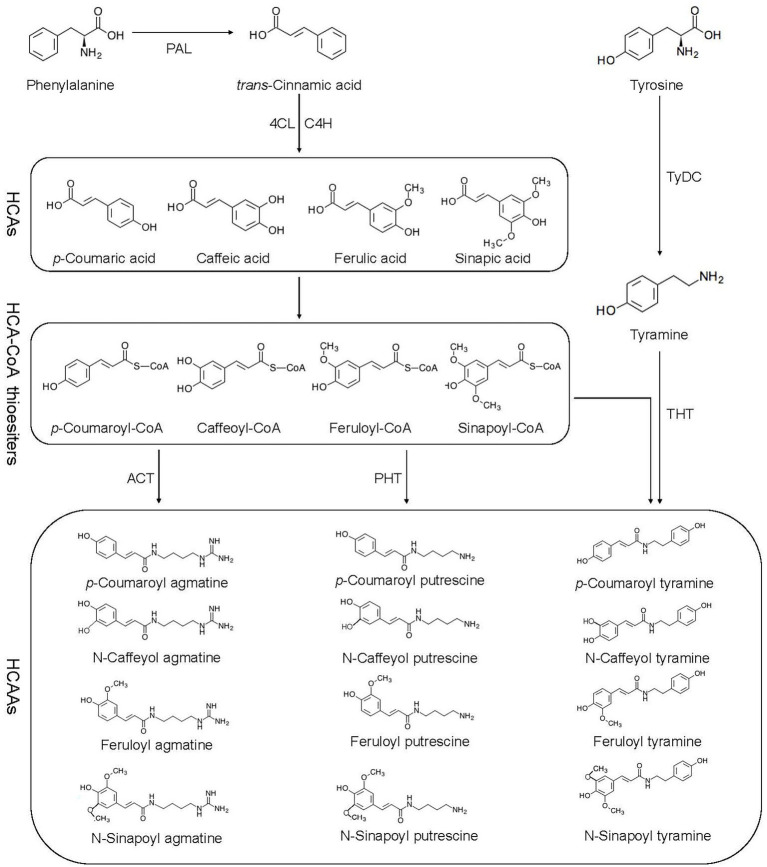
Biosynthetic pathway of HCAAs. HCAAs, hydroxycinnamic acid amides; HCAs, hydroxycinnamic acids; PAL, phenyl alanine ammonia lyase; C4H, cinnamate 4-hydroxylase; 4-CL, 4-coumarate: CoA ligase; ACT, agmatine coumaryl transferase; TyDC, tyrosine decarboxylase; THT, tyramine hydroxycinnamoyl transferase; and PHT, putrescence hydroxycinnamoyl transferase.

Flavonoids, hydroxycinnamic acid esters, hydroxycinnamic acid amides, the precursors of lignin, lignans, and tannins are end products of PPP (Gray et al., 2012). HCAAs are the products of an important branch of the PPP, in which phenylalanine ammonia lyase (PAL), cinnamic acid 4-hydroxylase (C4H), and 4-coumaric acid-CoA ligase (4CL) are all involved (Gray et al., 2012; Figure 1). The PPP begins with the deamination of phenylalanine by PAL to trans-cinnamic acid or cinnamate, and then catalyzed by 4CL (Hahlbrock and Scheel, 1989). PAL directs metabolic flow from the shikimate pathway to the various branches of PPP by catalyzing phenylalanine to trans-cinnamic acid (Zhang and Liu, 2015). The second reaction is catalyzed by C4H known to catalyze the hydroxylation of trans-cinnamic acid to *p*-coumaric acid (4-hydroxycinnamic acid; Schilmiller et al., 2009). The 4CL catalyzes the third step of PPP, forms *p*-coumaroyl-CoA in an ATP-dependent manner (Gui et al., 2011). The condensation of CoA derivative of *p*-coumaric acid (thioester *p*-coumaroyl-CoA) and the amine tyramine is catalyzed by hydroxycinnamoyl-CoA:tyramineN-hydroxycinnamoyltransferase (THT), and putrescine hydroxycinnamoyl transferase (PHT) further activates the synthesis of *p*-coumaroyltyramine and *p*-coumaroylputrescine (Pushpa et al., 2014). Agmatine coumaryl transferase (ACT) catalyzes the last step in the biosynthesis of the HCAAs, where *p*-coumaroylagmatine and feruloylagmatine are generated (Muroi et al., 2009). With the action of tyrosine decarboxylase (TyDC), tyrosine is further converted into tyramine (Von Roepenack-Lahaye et al., 2003).

## HCAAs Pathway Is Involved in Plant Disease Resistance

**Figure 2:**
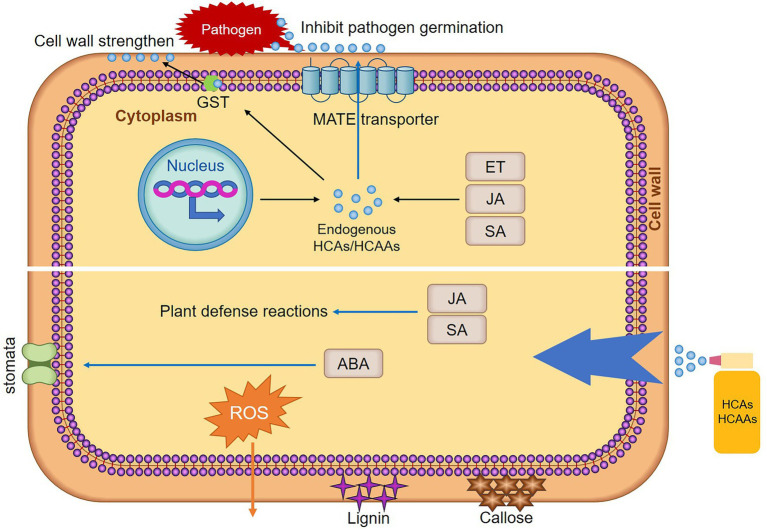
Mechanisms of HCAs/HCAAs enhancing plant resistance. After pathogen infection, elevation of plant endogenous hormones stimulates the production of HCAs/HCAAs. Rapid transcriptional reprogramming of genes encoding biosynthetic enzymes for HCAAs is one of the mechanisms of plant defense responses. Glutathione S-transferase (GST) may act as an amide carrier protein for HCAAs translocation to the plasma membrane, then deposited on the cell wall. Under the action of MATE, HCAAs move to the leaf surface, thereby inhibiting spore germination. Exogenous HCAs/HCAAs treatment stimulated a series of plant immune responses, such as plant hormones (JA, SA, and ABA) levels are elevated, SA and JA stimulate resistance responses in plants. HCAs/HCAAs stimulated stomata opening and closing, which may be achieved by controlling the content of ABA. HCAs/HCAAs also stimulate the production of lignin, callose, and ROS. The stimulation of these immune responses increases the resistance of plants to pathogens.

Hydroxycinnamic acid amides are conjugated PA, such as cinnamic acid, coumaric acid, caffeic acid, ferulic acid, and sinapic acid that form acylated PA (El-Seedi et al., 2012). This kind of secondary metabolites have been reported to protect plant cell against pathogen invasion by strengthening cell walls or act as antimicrobial agents directly (Campos et al., 2014). Meanwhile, the accumulation of HCAAs in plants contributes to the induction of plant hypersensitive responses (HR) in response to pathogen challenge (Walters, 2003). Since the first demonstration of *p*-coumaroyl- and feruloyl-2-hydroxyputrescine accumulation in leaves of rust-infected wheat (Samborski and Rohringer, 1970). Multiple studies have shown that HCAAs accumulated due to infection by pathogens. In wheat, untargeted metabolomic and proteomic analyses indicated that HCAAs were the major factor influencing *Fusarium graminearum* resistance (Gunnaiah et al., 2012). HCAAs synthesized as a result of *F. graminearum* infection were observed regardless of susceptibility, but occurred at different times after infection (Whitney et al., 2022). In the incompatible interaction between wheat and stripe rust, the HCAAs pathway was strongly induced, and *p*-coumaroyl agmatine was significantly increased (Liu et al., 2022). Significantly increased levels of sinapic acid and ferulic acid in wheat after infestation with stripe rust (Atta et al., 2020). In maize, HCAAs pathway was strongly induced after *Puccinia sorghi* infection (Kim et al., 2021). In cocoa-*phytophthora* spp., leaves of the tolerant genotype were found to accumulate dramatically higher levels of clovamide and several other HCAAs compared to the susceptible (Knollenberg et al., 2020). In potato, studies have suggested that HCAAs can be used as biomarker metabolites for late blight resistance and black dot (Pushpa et al., 2014). In banana, nematode-resistant varieties have very marked increases in *p*-coumaric, ferulic, and sinapic acid content compared to susceptible varieties (Vaganan et al., 2014). HCAAs were significantly increased in *Arabidopsis* after *Sclerotinia sclerotiorum* infection (Chen et al., 2021). Furthermore, plant growth-promoting rhizobacteria was shown to affect root HCAAs content in rice (Valette et al., 2020). The above reports fully demonstrated that the accumulation of HCAAs improves the resistance of plants to pathogens. Plant endogenous HCAs/HCAAs defend against infection by pathogens, and exogenous application of HCAs/HCAAs to resist the infection of pathogens by stimulating the production of plant immune responses (Figure 2).

### Transcriptional Responses of HCAAs Pathway Related Genes in Plant Defense

Rapid transcriptional reprogramming of genes encoding biosynthetic enzymes for protective secondary metabolites (such as HCAAs) is one of the mechanisms of plant defense responses. During *Alternaria brassicicola* infection, the expression of *AtACTs* (*ATT3G03480*, *AT5G01210*, *AT5G39050*, *and AT5G61160*) in *Arabidopsis* was rapidly induced (Muroi et al., 2009). In resistant varieties of potato, relative expression of *StTyDC*, *StTHT*, *St4CL*, and *StPHT* was induced by *Solanum tuberosum* infection (Pushpa et al., 2014). The transcriptional alterations of key genes affect HCAAs levels in pathogen-infected plants, leading to changes in plant defense responses. The *Atact* mutant was susceptible to infection by *A. brassicicola* and *p*-coumaroylagmatine content of *Atact* mutant was reduced (Muroi et al., 2009). Likewise, wheat *TaACT*-knockout plants exhibited that *p*-coumaroylagmatine content was significantly reduced and susceptibility to *F. graminearum* was increased (Kage et al., 2017a). In contrast, overexpression of *SlTHT* in tomato increased the accumulation of tyramine and octopamine derivatives and enhanced tomato resistance against *P. syringae* (Campos et al., 2014). Additionally, ectopic expression of *AtACT* increased the resistance of torenia plants to *Botrytis cinerea* (Muroi et al., 2013). In potato, simultaneous overexpression of *AtACT* and *Arabidopsis DETOOXIFICATION18* (*AtDTX18*) genes gave plants the ability to synthesize *p*-coumarin and export it to the leaf surface, so that increased the accumulation of HCAAs on plant leaf surface and reduced spore germination of *Phytophthora infestans* (Dobritzsch et al., 2016; Figure 2). Stable expression of N-caffeoyl-L-3,4-dihydroxyphenylalanine hydroxycinnamoyl transferase (HDT) in alfalfa resulted in foliar accumulation of p-coumaroyl- and feruloyl-L-Tyr and transient expression of HDT in *Nicotiana benthamiana* resulted in the production of caffeoyl-L-Tyr (Sullivan and Knollenberg, 2021). The above studies showed that key genes in the HCAAs pathway play a role in plant disease resistance by controlling the synthesis of HCAAs. Key genes in the HCAAs pathway have great potential for application in disease resistance breeding.

### Antimicrobial Activity of HCAs/HCAAs

Hydroxycinnamic acids and their derivatives also show antimicrobial activity. As shown in Table 1, the inhibitory effect of HCAs/HCAAs on microorganisms involves at least two processes: direct antimicrobial activity and strengthening of secondary cell walls (Roumani et al., 2021). Natural compounds, such as caffeic acid, syringic acid, *p*-coumaric acid, and ferulic acid have strong inhibitory effect on pathogenic fungi (Hassan et al., 2021). HCAAs inhibits the growth of *Aspergillus niger*, *F. aureus*, and *Penicillium verruciformis* (Kyselka et al., 2018). Trans-cinnamic acid, ferulic acid, and *p*-coumaric acid can inhibit the growth of *Colletotrichum acutatum* (Roy et al., 2018). Ferulic acid, the most abundant HCAs in the plant kingdom, is an ester linked to arabinose (Mathew and Abraham, 2006). Ferulic acid inhibited the growth of *F. oxysporum* at high concentrations and affected the activity of hydrolases associated with pathogenicity (Wu et al., 2010). Ferulic and rho-coumaric acids reduced *Alternaria* growth *in vivo* and black spot in stored fruits (Yadav et al., 2021). Ferulic acid leads to irreversible changes in cell membrane properties (charge, intracellular and extracellular permeability, and physicochemical properties) through hydrophobicity changes, reduction in negative surface charges, and local rupture of the cell membrane or pore formation, resulting in the leakage of essential intracellular components of pathogenic bacteria (Borges et al., 2013). Exogenous caffeic acid enhanced apple resistance to *B. cinerea* (Zhang et al., 2020). Caffeic acid can damage the membrane structure of *R. solanacearum* cells, resulting in thinning of the cell membrane and irregular intracellular voids. In addition, caffeic acid can also inhibit biofilm formation by inhibiting the expression of *lecM* and *epsE* genes. Exogenous caffeic acid also effectively activated peroxidase and PAL (Li et al., 2021b). Sinapic acid was chemically studied as a cinnamic acid derivative and also inhibited the growth, conidial germination of *F. oxysporum* and reduced the activity of pathogenic enzymes at high doses (Wu et al., 2009). *In vivo* test, changes in the levels of phenolic compounds in infected plants and their antifungal activity for against *Verticillium dahliae* strongly suggested that *p*-coumaric acid was involved in the natural defense or resistance mechanisms of plant (Baidez et al., 2007). Exogenously *p*-coumaric acid increased chitinase activity in leaves and β-1,3-glucanase activity in roots, thereby enhancing watermelon resistance to *F. oxysporum* (Ren et al., 2016). The antibacterial mechanism of *p*-coumaric acid includes two aspects: disrupts bacterial cell membranes and binds to bacterial genomic DNA to inhibit cell function, ultimately leading to cell death (Lou et al., 2012). However, *p*-coumaric acid did not affect the integrity of the cell wall and plasma membrane of *B. cinerea*, nor did it produce oxidative stress (Morales et al., 2017). HCAAs have been shown against bacteria by regulating the expression of the type III secretion system (T3SS). For example, *p*-coumaric acid inhibited the expression of T3SS gene of the plant pathogen *Dickeya dadantii* (Li et al., 2009). However, ferulic acid may mimic host conditions, thereby activating T3SS expression (Zhang et al., 2017). High concentrations of HCAs can inhibit the growth of *R. solanacearum* in the medium. However, *R. solanacearum* can protect itself from HCAs toxicity by degrading low concentrations of HCA as the sole carbon source (Lowe et al., 2015). Chitosan treatment of wheat increased the content of *p*-coumaric acid, ferulic acid, and sinapic acid in leaves and enhanced resistance to *F. graminearum* (Reddy et al., 1999). The above studies have proved that HCAs can inhibit the growth of pathogenic fungi and spore germination, and HCAs can inhibit pathogenic bacteria by damaging the cell membrane and DNA of pathogenic bacteria. Pathogens have also evolved mechanisms to reduce the harm of HCAs/HCAAs, and this aspect will also be the focus of research.

**Table 1:** Antimicrobial activity of various hydroxycinnamic acids (HCAs)/hydroxycinnamic acid amides (HCAAs) and their mechanisms.

Host	Pathogen	Disease name	Compounds	Mechanism	References
*Solanum lycopersicum*	*Pseudomonas syringae*	Bacterial speck of tomato	*p*-coumaroyltyramine, feruloyltyramine	SA, PR gene	Campos et al., 2014
*Torenia fournieri Linden*	*Botrytis cinerea*	_	*p*-coumaroylagmatine	Inhibit the germination and development of conidial germ tubes	Muroi et al., 2013
*Solanum tuberosum*	*Phytophthora infestans*	Potato late blight disease	*p*-coumaroylagmatine	Inhibit spore germination	Dobritzsch et al., 2016
*Fragaria x ananassa* Duch	*Colletotrichum acutatum*	Strawberry anthracnose	trans-cinnamic acid, ferulic acid, and p-coumaric acid	Inhibit fungal growth	Roy et al., 2018
*Citrullus lanatus*	*Fusarium oxysporum* f. sp. *niveum*	Watermelon fusarium wilt	Ferulic acid	Inhibit spore germination	Wu et al., 2010
*Diospyros kaki* L. var	*Alternaria*	black spot disease	Ferulic, and rho-coumaric acids	Reduce growth	Yadav et al., 2021
_	*Escherichia coli*, *Pseudomonas aeruginosa, Staphylococcus aureus*, and *Listeria monocytogenes*		Ferulic acids	Changes in membrane properties	Borges et al., 2013
*Malus pumila* Mill.	*Botrytis cinerea*	Apple gray mold	Caffeic acid	Activation of different branches of the phenylpropanoid metabolism pathway	Zhang et al., 2020
*Nicotiana tobaccum*	*Ralstonia solanacearum*	Tobacco bacterial wilt	Caffeic acid	Damaged the membrane structure and promote the accumulation of lignin	Li et al., 2021b
_	*Fusarium oxysporum* f. sp. *niveum*		Sinapic acid	Inhibit the growth and conidial germination	Wu et al., 2009
*C. lanatus*	*Fusarium oxysporum* f. sp. *niveum*	Watermelon fusarium wilt	*p*-coumaric acid	Increased β-1,3-glucanase activity	Ren et al., 2016
_	*Verticillium dahliae*		*p*-coumaric acid	_	Baidez et al., 2007
_	*S. aureus*, *Streptococcus pneumoniae Bacillus subtilis*, *E. coli*, *Shigella dysenteriae* and *Salmonella typhimurium*		*p*-coumaric acid	Disrupted cell membranes and binding to bacterial genomic DNA	Lou et al., 2012
_	*Botrytis cinerea*	_	*p*-coumaric acid	Retard the germination of conidia	Morales et al., 2017
_	*Dickeya dadantii*	_	*p*-coumaric acid	Manipulating the expression of the T3SS	Li et al., 2009
*S. tuberosum*	*P. infestans*	Potato late blight disease	N-4-coumaroyl- and N-Feruloyltyramine	Cell wall reinforcement	Schmidt et al., 1998
*Arabidopsis thaliana*	*Erwinia carotovora carotovora*		Coumaroyl tyramine and coumaroyl tryptamine	Increased the induction of callose deposition	Macoy et al., 2022
*Brassica napus*	*Xanthomonas campestris* pv. *campestris*		*p*-coumaric acid	Primed the JA-signaling-mediated induction of phenylpropanoid biosynthesis	Islam et al., 2019

### HCAAs Strengthen Plant Cell Walls

Polyamines and aromatic amines bind to HCAs phenolic resins, leading to the formation of HCAAs-molecules that have the ability to confer antimicrobial activity and potentially deposit into cell walls (Zeiss et al., 2021). HCAAs in the cytosol may be transported to different vesicles and then to the plasma membrane, allowing the deposition of HCAAs into the cell wall. Glutathione S-transferase (GST) may act as an amide carrier protein for HCAAs translocation to the plasma membrane (Macoy et al., 2015; Figure 2). HCAAs constitute the polyaromatic domains of suberin. These polymers increased the thickness of the cell wall, limit the spread of pathogens, and act as antifungals. As a plant defense response, HCAAs require the deposition of amide conjugates in the cell wall to reduce fungal pathogen penetration and infection (Graca, 2010). HCAAs, such as coumaroylputrescine, feruloylputrescine, cinnamoyltyramine, cis-*p*-coumaroylagmatine, feruloylagmatine, coumaroylserotonin, caffeoylserotonin, and feruloylserotonin were proven to increase cell wall thickness in wheat to resist *F. graminearum* (Gunnaiah et al., 2012). Deposition of HCAAs was thought to form a barrier against pathogens by reducing cell wall digestibility (Facchini et al., 2002). In date palm-resistant cultivars, cell wall-bound phenols (*p*-coumaric acid, ferulic acid, and sinapic acid) were found to strongly reduce hyphal growth and cell wall-degrading enzymes of *F. oxysporum* production (El Modafar and El Boustani, 2001). Fungal infection of potato cell cultures and leaves has been reported to trigger the incorporation of *p*-coumaroyltyramide and feruloyltyramide into the cell wall (Schmidt et al., 1998). The two main HCAs, *p*-coumaric acid and ferulic acid, were present in the complex cell walls of oat husks and act as crosslinkers between lignin and polysaccharides or between polysaccharides. Therefore, they inhibited the biodegradation of the cell wall by microorganisms (Yu et al., 2004). HCAAs prevent the infection of pathogens by strengthen the cell wall and reduced the degradation of the cell wall.

### HCAs/HCAAs Regulate Lignin and Callose Deposition

Lignin and callose deposition are two late immune responses that enhance plant cell walls. Lignin is mainly deposited in the secondary cell walls of plants (Zhao and Dixon, 2011). During pathogens infection, lignin is deposited and acts as a physical barrier to limit the spread of pathogens (Lee et al., 2019). Lignin deposition around pathogen penetration sites was found to increase potato resistance to *P. infestans* (Sorokan et al., 2018). Tobacco with lower total lignin content shows tobacco susceptibility to blackleg and bacterial wilt (Ma et al., 2018). HCAAs also affect lignin content (Figure 2). For example, exogenously applied *p*-coumaric acid, caffeic acid, ferulic acid, and sinapic acid were directed into the PPP, resulting in the overproduction of lignin and its main monomers (Lima et al., 2013). The perception of pathogen or microbe-associated molecular pattern molecules by plants triggers a basal defense response analogous to animal innate immunity and was defined partly by the deposition of the glucan polymer callose at the cell wall at the site of pathogen contact (Clay et al., 2009). Deposition of callose prevented powdery mildew hyphae from entering epidermal cells (Ellinger et al., 2014). Exogenous application of coumaroyltyramine and coumaroyltryptamine increased the induction of callose deposition (Macoy et al., 2022). These findings also indicate that HCAAs can resist the infection of pathogens by enhancing the synthesis of lignin and the deposition of callose.

### HCAs/HCAAs Contribute to Stomatal Immunity

Stomatal is a dynamic and captivating system that opens or closes in response to external and internal cues. As part of the innate immune system, stomatal closure can limit bacterial invasion and act as a barrier against bacterial infection. Upon perception of pathogens, plants can rapidly close their stomates to restrict pathogen entry into internal tissue (Melotto et al., 2006). Stomates of silenced *NbGCN4* and *AtGCN4* were open during pathogen infection, leading to compromised disease resistance in both host and nonhost (Kaundal et al., 2017). Arabidopsis *bzip59* mutant was partially impaired in stomatal closure induced by *Pst* DC3000 and was more susceptible to *Pst* DC3000 infection (Song et al., 2022). The application of exogenous compounds also affected the opening and closing of stomatal, such as HCAAs, with sinapic acid leading to considerable closure, while ferulic acid stimulated wider openings (Plumbe and Willmer, 1986; Figure 2). However, recent studies have shown that low concentrations of ferulic acid significantly inhibited stomatal opening, stomatal opening rate, stomatal length and width (Fu et al., 2019). This may be due to the different concentrations of ferulic acid, resulting in different results on stomates. Plant endogenous abscisic acid (ABA) level was increased after ferulic acid treatment (Holappa and Blum, 1991), and may regulated the opening and closing of stomatal by regulating the biosynthesis of ABA.

### Role of HCAs/HCAAs in Reactive Oxygen Species

Hydroxycinnamic acid amides are formed by conjugation of amines with HCAs. Free-form PA may play two conflicting roles in regulating cellular ROS: as a source of ROS biosynthesis and a free radical scavenging compound. The catabolism of PA leads to increased intracellular and extraplasmic H_2_O_2_ concentrations (Zeiss et al., 2021). Production of ROS, a hallmark of successful identification of infection and activation of plant defenses, is one of the early ways in host plant defense strategies (Qi et al., 2017). It achieves plant immunity by eliminating damaged host cells, and limits further pathogen infection. ROS may be involved in signaling in plants against pathogens, enhanced resistance to stressors, and downstream cellular defense-related genes that limit infection through pathogen death (Suzuki et al., 2011). Furthermore, ROS have also been postulated as key molecules involved in the initiation of HR. Accumulation of HCAAs also plays an important role in plant HR. However, HCAAs also have antioxidant activity (Xiang et al., 2019). Studies have shown that the oxidative burst in cucumber roots occurs under the influence of ferulic acid and *p*-coumaric acid (Politycka and Bednarski, 2004; Figure 2). Ferulic acid concentration can lead to accumulation of ROS, and after exogenous ferulic acid stimulation, the induced genes were involved in cell wall formation, chemical detoxification, secondary metabolism, and signal transduction (Chi et al., 2013). In addition, the accumulation of ferulic acid also enhances the antioxidant capacity of plants (Zhang et al., 2022). It remains to be seen how plants are both contradictory and unified in this regard.

### Accumulation of HCAs/HCAAs Is Associated With Plant Hormones

Disease resistance mediated by plant hormones, such as SA, ethylene (ET), and JA can be induced by different exogenous signals in different plants to resist different types of pathogens, which is the basic signaling pathway of plant disease resistance (Feys and Parker, 2000). These plant hormones regulated the synthesis of compounds. JA regulated the biosynthesis of many secondary metabolites, including HCAAs (Li et al., 2021a). The accumulation of *p*-coumaroylagmatine in leaves was higher at 48 h after JA treatment, and reached the highest level at 48 h after JA/ET combined treatment. These results proved that HCAAs accumulation was induced by JA and ET signals (Li et al., 2018). It has also been demonstrated that pathogen-induced ET production was essential for synthesis of HCAAs, whereas SA was also a key signal for initiating plant defense responses, not required for this response (Zacares et al., 2007). Similarly, it has also been reported that ET produced after bacterial infection was essential for the accumulation of HCAAs, and with ET as a signal to respond to pathogen attack, SA was not involved in the accumulation of HCAAs (Lopez-Gresa et al., 2011). These studies demonstrated the role of ET and JA in intracellular signaling cascades, leading to the accumulation of secondary compounds and ultimately the induction of plant resistance. However, studies have reported accumulation of HCAAs (coumaryltyramide and feruloyltyramide) accompanied by elevated SA levels and pathogenesis-related genes induction (Campos et al., 2014). The production of ferulic acid, *p*-coumaric acid, and sinapic acid was stimulated by exogenous SA (Zhang et al., 2021). In turn, exogenous HCAs can enhance these plant hormones changes and improve plant resistance (Figure 2). The contents of caffeic acid, ferulic acid, and *p*-coumaric acid were increased in Methyl jasmonate and SA treated plants (Napoleao et al., 2017). JA content and expression of signaling genes (*COI1* and *PDF1.2*) were increased in *p*-coumaric acid-pretreated plants, and exogenous *p*-coumaric acid triggered JA signaling-mediated induction of phenylpropanoid biosynthesis, which elicited disease resistance to black rot disease in *Brassica napus* (Islam et al., 2019). These studies demonstrated that HCAAs and plant hormones regulate each other and participate in the process of plant disease resistance together.

## Regulation of HCAAs Biosynthesis

The spatial, temporal and induced formation of secondary metabolites and transcripts of corresponding biosynthetic genes are tightly regulated at different levels. Transcription factors (TFs) usually regulate the transcription of multiple biosynthesis genes in a pathway, which makes them attractive tools for improvement of the production of secondary metabolites (Zhou and Memelink, 2016). TFs can integrate internal and external signals to regulate genes expression, thereby controlling the specific accumulation of secondary metabolites (Yang et al., 2012). Many studies have demonstrated that TFs can regulate HCAAs pathway.

StWRKY1 can directly bind to the promoter encoding the HCAAs biosynthesis genes. After *StWRKY1* gene silencing, the abundance of HCAAs and potato resistance to late blight were reduced (Yogendra et al., 2015). Silencing of *StNACA3* and *StMYB8* altered late blight resistance in the silenced plants by significantly increasing pathogen biomass and reducing the contents of HCAAs and flavonoid glycosides (Yogendra et al., 2017). *HvWRKY23* also exhibits the same characteristics, which in turn increases barley resistance to Fusarium head blight by regulating HCAAs synthesis (Karre et al., 2019). Induced expression of the MYB caused accumulation of ferulic acid and enhanced resistance to both fungal and bacterial pathogens in plant (Kishi-Kaboshi et al., 2018). Overexpression of the petunia MYB transcript factor, *ODORANT1* (*ODO1*), combined with expression of a feedback-insensitive *E.coli* 3-deoxy-D-arabino-heptulosonate 7-phosphate synthase (AroG), altered the levels of multiple primary and secondary metabolites in tomato fruit, boosting levels of multiple secondary metabolites, including *p*-coumaric acid and ferulic acid (Xie et al., 2016). *AtMYB99*, a putative ortholog of the petunia floral scent regulator *ODO1*, controls the exclusive production of HCAAs (Battat et al., 2019). After *TaWRKY70* gene silencing, not only confirmed the weakening of resistance to *F. graminearum*, but also reduced the expression of downstream resistance genes *TaACT*, *TaDGK* and *TaGLI1*, as well as the content of coumaroylagmatine and coumaroylputrescine (Kage et al., 2017b). Despite of numerous studies have shown that TFs regulate HCAAs biosynthesis, more studies are needed to further elucidate the relationship between TFs and HCAAs, as well as in plant–pathogen interactions.

In addition, HCAAs were also affected by UDP-glycosyltransferases (UGTs). UGTs are responsible for the glycosylation of small molecule compounds. Glycosylation can affect the homeostasis of these substances by altering chemical activity, degradation, and/or localization of compounds (Bowles et al., 2005). Glycosylation modulates the biological activity of small molecules and often results in their inactivation. *NbUGT73A24* and *NbUGT73A25* can glycosylate N-feruloyl tyramine and ferulic acid derivatives (ethyl 4-hydroxy-3-methoxy-cinnamate, trans-ferulic acid, 4-coumaric acid, caffeic acid, N-caffeoyl O-methyltyramine, N-4-trans-coumaroyl tyramine, and N-trans-feruloyl-tyramine), overexpression of both genes in *Nicotiana benthamiana* produced clear lesions on the leaves and led to a significantly reduced content of pathogen-induced plant metabolites (Sun et al., 2019). A recent study showed that *UGT73C7* can glycosylate *p*-coumaric acid and ferulic acid glycosylation activity of *UGT73C7* resulted in the redirection of phenylpropanoid metabolic flux to the biosynthesis of HCAs and coumarins, thereby affecting the immune response of plants (Huang et al., 2021). *P*-coumaric acid and ferulic acid are precursors of various metabolites, and the glycosylation of *p*-coumaric acid and ferulic acid by *UGT73C7* significantly affects the metabolism of phenylpropanes. These evidences suggested that glycosylation can simultaneously affect the abundance of HCAs/HCAAs, thereby regulating plant disease resistance.

## Challenges Remained and Future Directions

Metabolites of HCAAs pathway were involved in various stress responses, play an important role in plants and can also directly inhibit microorganisms (Figure 2). It is necessary to conduct more in-depth studies on metabolites. The information from this review focuses on the induction of plant defense responses to biotic stress by HCAAs pathway, illustrating the directly inhibition of microorganisms by HCAs/HCAAs, the defense responses induced by exogenous HCAs/HCAAs in plants, and the regulation of HCAAs biosynthesis. The study helps us to understand the possible functions of HCAAs pathway during plant–pathogen interactions. HCAs/HCAAs have the potential to be developed into plant antagonists, and will also be a hotspot. Metabolomics is emerging as a new tool for understanding plant–pathogen interactions. In the future, there will be some new insights into how HCAs/HCAAs can improve the defense response of plants. Moreover, there is a rising trend in the development and application of molecular marker assays for gene mapping and discovery in field crops and trees (Rasheed et al., 2017). Resistance genes in the HCAAs pathway and genes of regulated HCAAs biosynthesis also have great application potential in molecular breeding. Varieties with high hydroxycinnamic acid concentrations can be used to produce dietary supplements or all-natural food additives, while enhancing resistance to biotic and abiotic stresses during the growing season and during grain storage.

## Author Contributions

All authors listed have made a substantial, direct, and intellectual contribution to the work and approved it for publication.

## Funding

This research was financially supported by the National Natural Science Foundation of China (32001975), and the Fundamental Research Funds for the Central Universities (SWU-KT22057).

## Conflict of Interest

The authors declare that the research was conducted in the absence of any commercial or financial relationships that could be construed as a potential conflict of interest.

## Publisher’s Note

All claims expressed in this article are solely those of the authors and do not necessarily represent those of their affiliated organizations, or those of the publisher, the editors and the reviewers. Any product that may be evaluated in this article, or claim that may be made by its manufacturer, is not guaranteed or endorsed by the publisher.
